# Microscale Characterization and Trace Element Distribution in Bacteriogenic Ferromanganese Coatings on Sand Grains from an Intertidal Zone of the East China Sea

**DOI:** 10.1371/journal.pone.0119080

**Published:** 2015-03-18

**Authors:** Linxi Yuan, Liguang Sun, Danielle Fortin, Yuhong Wang, Xuebin Yin

**Affiliations:** 1 School of Earth and Space Sciences, University of Science and Technology of China, Hefei 230026, China; 2 Jiangsu Bio-Engineering Research Centre on Selenium, Suzhou 215123, China; 3 Advanced Lab for Selenium and Human Health, Suzhou Institute for Advanced Study, University of Science and Technology of China, Suzhou 215123, China; 4 Department of Earth Sciences, University of Ottawa, Ontario, K1N 6N5, Canada; 5 National Institute of Health, Bethesda, Maryland 20892, United States of America; The University of Akron, UNITED STATES

## Abstract

An ancient wood layer dated at about 5600 yr BP by accelerator mass spectrometry (AMS) ^14^C was discovered in an intertidal zone of the East China Sea. Extensive and horizontally stratified sediments with black color on the top and yellowish-red at the bottom, and some nodule-cemented concretions with brown surface and black inclusions occurred in this intertidal zone. Microscale analysis methods were employed to study the microscale characterization and trace element distribution in the stratified sediments and concretions. Light microscopy, scanning electron microscopy (SEM) and backscattered electron imaging (BSE) revealed the presence of different coatings on the sand grains. The main mineral compositions of the coatings were ferrihydrite and goethite in the yellowish-red parts, and birnessite in the black parts using X-ray powder diffraction (XRD). SEM observations showed that bacteriogenic products and bacterial remnants extensively occurred in the coatings, indicating that bacteria likely played an important role in the formation of ferromanganese coatings. Post-Archean Australian Shale (PAAS)-normalized middle rare earth element (MREE) enrichment patterns of the coatings indicated that they were caused by two sub-sequential processes: (1) preferentially release of Fe-Mn from the beach rocks by fermentation of ancient woods and colloidal flocculation in the mixing water zone and (2) preferential adsorption of MREE by Fe-Mn oxyhydroxides from the seawater. The chemical results indicated that the coatings were enriched with Sc, V, Cr, Co, Ni, Cu, Zn, Ba, especially with respect to Co, Ni. The findings of the present study provide an insight in the microscale features of ferromanganese coatings and the Fe-Mn biogeochemical cycling during the degradation of buried organic matter in intertidal zones or shallow coasts.

## Introduction

In natural environments, iron oxide minerals included poorly ordered hydrous ferric oxide (HFO) minerals, such as ferrihydrite (Fe_5_HO_8_·4H_2_O), and more crystalline forms, such as goethite (α-FeOOH), lepidocrocite (γ-FeOOH), hematite (α-Fe_2_O_3_), and magnetite (Fe_3_O_4_) [1]. Iron oxide minerals accumulated in sediments and played an important role in the sorption of trace elements, heavy metals, and nutrients [2–3]. Particularly, biogenic HFO were considered to be dominant sorbents of dissolved metals in aquatic environments because of their broad distribution and reactive surface properties [4–5]. Mn oxide minerals, such as todorokite ((Na, Ca, K)_2_(Mn^4+^, Mn^3+^)_6_O_12_·3–4.5H_2_O), birnessite ((Ca, Na)_0.5_ (Mn^4+^, Mn^3+^)_2_O_4_·1.5H_2_O), and vernadite ((Mn^4+^, Fe^3+^, Ca, Na) (O, OH)_2_·nH_2_O), were highly reactive mineral phases to control the distribution and bioavailability of many toxic and essential elements, which played important roles in elemental biogeochemical cycles in nature [6–7].

Iron and manganese oxyhydroxides in marine and freshwater sediments were often biogenic indicators [6, 8–12]. Iron-oxidizing bacteria, especially *Gallionella ferruginea* (G) and *Leptothrix ochracea* (L), were known to be key players in the formation of iron oxyhydroxides in aquatic environments [13]. Bacteriogenic iron oxyhydroxides (such as ferrihydrite) could be transformed to goethite by enhanced proton activity in the vicinity of cell surface [14], as observed in anoxygenic phototrophic Fe-oxidizing bacteria [15]. Biological processes were shown to be responsible for Mn(II) oxidation [16–18], and it was hypothesized that biological Mn(II) oxidation dominated in the natural environment [7, 11–12, 19–20].

Ferromanganese coatings on sand grains were typically composed of fine-grained material and poorly crystalline minerals, which were of environmental significance for bioremediation [6, 21–23]. Due to the difficulties in sample preparation and the variability in crystallinity of the coating constituents [24–27], few mineralogical and geochemical studies on these coatings have been performed at microscale. As a result, the formation mechanisms of these coatings were still poorly understood [24–29]. Rare earth element (REE) patterns usually provided useful information on the origin of natural samples and the environment where they have formed [26, 30]. Although REEs in marine ferromanganese concretions or crust from seafloor have been extensively studied [31–32], few studies were performed on ferromanganese samples in the intertidal zone area.

In the present study, extensive ferromanganese coatings on sand grains were discovered from an intertidal zone of East China Sea. The coatings were clearly distinguishable by their color: a yellowish-red part and a black part. In order to characterize the microscale features of the coatings, X-ray powder diffraction (XRD), scanning electron microscopy (SEM)—energy dispersive X-ray spectrometer (EDS), and backscattered electron (BSE) imaging—X-ray mapping were used to identify the minerals, describe the micro-morphological characteristics, and determine the association among the various coating materials. Inductively coupled plasma—mass spectrometry (ICP-MS) was employed to quantify trace elements in the coatings, and examine the trace metal partitioning between iron-oxyhydroxide coatings and manganese-oxyhydroxide coatings. Furthermore, we discussed the biogeochemical processes for the formation of ferromanganese coatings.

## Background

**Fig 1:**
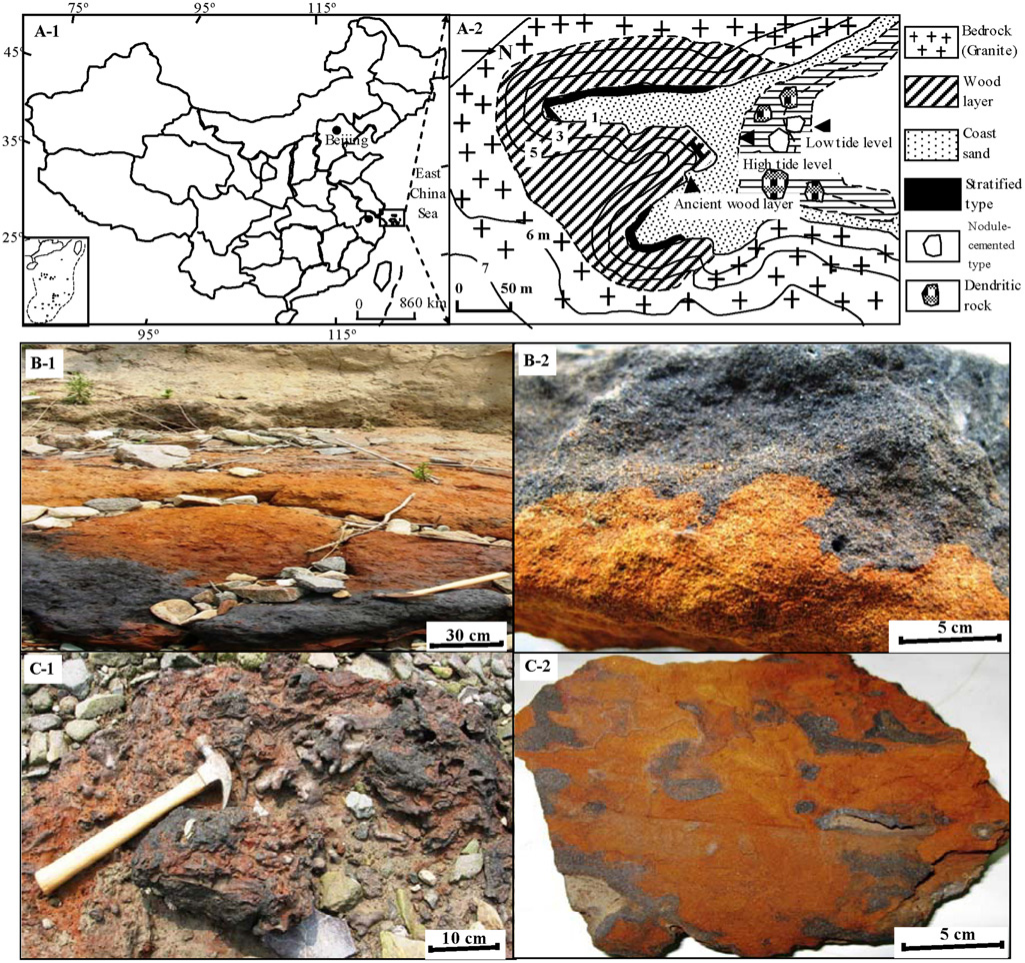
Study site located in intertidal zone, East China Sea (A-1, 2); Two typical samples, stratified type (S-type) (B-1, 2) and nodule-cemented type (N-type) (C-1, 2), were found in field. The photos of B-1 and C-1 were taken during field work, and those samples were cut in laboratory (B-2 and C-2).

**Fig 2:**
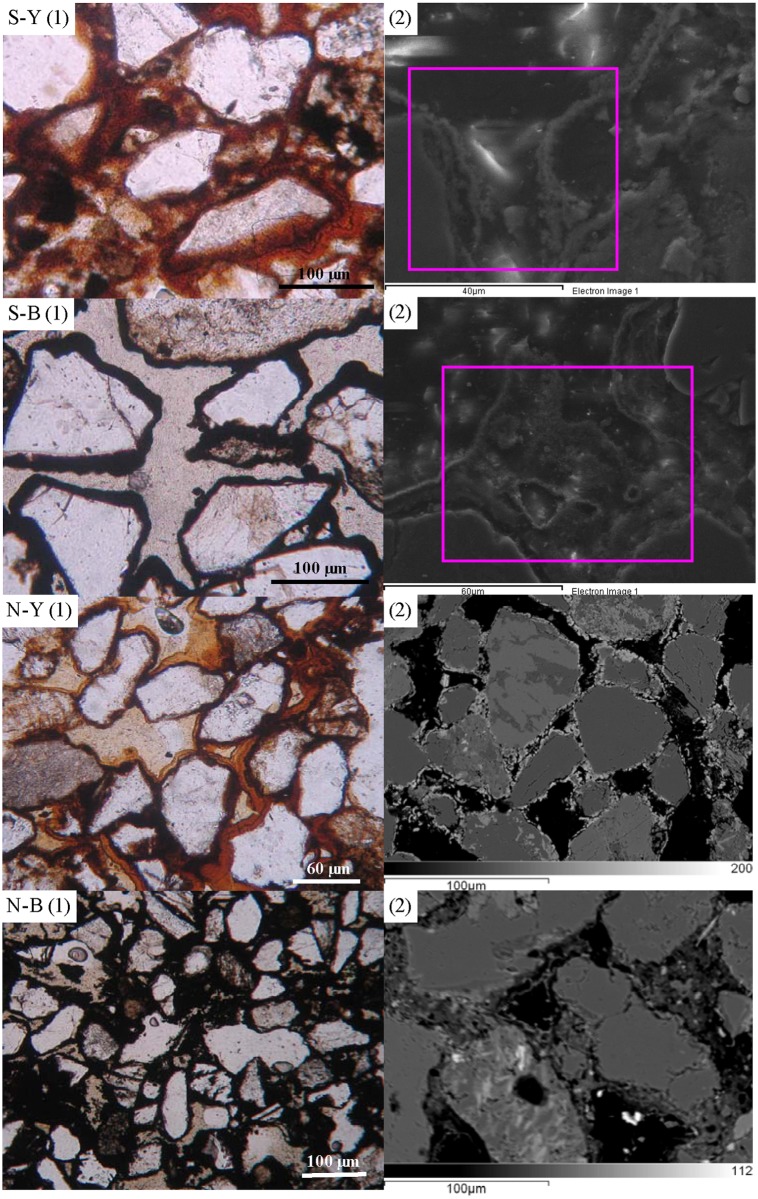
Plane polarized light (left), scanned electron (right upper two) and backscattered electron (right lower two) micrographs of epoxy-embedded thin sections of two typical samples. S-Y and S-B represented the yellowish-red part and the black part of S-type sample, respectively; N-Y and N-B for the yellowish-red part and the black part of N-type sample, respectively.

An ancient wood layer about 3.3 m thick and 500 m long, was discovered in an intertidal zone of Zhujiajian Island, Zhoushan Archipelago, East China Sea (Fig. 1A-1, 2). Previous studies conducted by our group revealed that because of the fermentation of ancient woods, acidic pH (pH = 2.60), low oxygen content (DO = 2.19 mg/L), and reducing (Eh = -148.8 mV) seepage water significantly accelerated the release of Fe and Mn from bedrocks into the intertidal zone [33]. Fresh bacteriogenic oxides (BIOS) were present near the ancient wood layer characterized by very high contents of Fe (41.54%) and Mn (0.51%), which were 7–25 and 17–25.5 times higher than those of weathering bedrocks [33–35]. Iron-oxidizing bacteria, such as *Leptothrix*-like sheaths and *Gallionella*-like stalks, were abundant in BIOS, and played important roles in Fe biomineralization in the present study site [35]. The ancient woods also provided abundant carbon source for sulfate-reducing-bacteria in the intertidal area with high content of chromium reducible sulfide (447.42 μmol/g) and unique δ^34^S (-2.9 ‰) in the beach mud [33].

## Materials and Methods

### 3.1 Ethics statement

This study was part of the approved scientific plan of Chinese Academy of Sciences (CAS). As this was a purely scientific study and the study site (N 29.94^o^, E 122.39^o^) was not a national park, a private land or any protected area of land or sea, no specific permits was needed. The field study did not involve endangered to protected species.

### 3.2 Sample collection and preparation

Sample names, description and analysis techniques were summarized in Table 1. Extensive and unconsolidated sediments were found near the ancient wood layer, and they were horizontally stratified with black color on the top and yellowish-red at the bottom (Fig. 1B-1, -2). In the nearby beach area, some nodule-cemented concretions with brown surface were found, and the diameter varied between 20–50 cm (Fig. 1C-1). In the transverse section of the concretion, some black nodules or tunnels, 1–5 cm in diameter, were interspersed in the yellowish-red substrates (Fig. 1C-2). Pieces of the stratified sample (S-type) (Fig. 1B) and the nodule-cemented concretion sample (N-type) (Fig. 1C) were cut with a chisel and collected in clean plastic bags. The black parts and the yellowish-red parts of S-type and N-type samples were separated and labeled as S-B, S-Y, N-B, and N-Y, respectively. To study the coating materials, gentle abrasion of the coatings from the sand grains in purified water was performed according to Penn et al. (2001) [24]. The resulted liquids were filtered through 0.20 μm films and the trapped materials on the films were freeze-dried at-80^o^C. Four subsamples were obtained and labeled as S-B-Coating, S-Y-Coating, N-B-Coating, and N-Y-Coating. Meanwhile, beach rock (BR), bacteriogenic iron oxides (BIOS) in seepage system, beach mud (BM), weathering profile including red soil (RS) and white soil (WS), seawater (SW) and groundwater (GW) were collected from the intertidal area for purpose of comparison. The S-B, S-Y, N-B and N-Y samples were embedded in epoxy resin. Once polymerized, the blocks were cut transversally using diamond saw, and fine polished to about 30 micrometer thick. The prepared thin sections were directly observed under plane polarized light by Olympus-BX51-P (Light Microscope).

**Table 1:** Key information for collected sample names, description and analysis techniques.

Name	Description	Analysis techniques
S-Y	Yellowish-red part of stratified type	Light microscopy, SEM-EDS, ESEM-X-Ray mapping
S-B	Black part of stratified type	Light microscopy, SEM-EDS, ESEM-X-Ray mapping
N-Y	Yellowish-red part of nodule-cemented concretion type	Light microscopy, BSE, SEM-EDS, EMPA-X-Ray mapping
N-B	Black part of nodule-cemented concretion type	Light microscopy, BSE, SEM-EDS, EMPA-X-Ray mapping
S-Y-Coating	Coatings abraded off yellowish-red part of stratified type	XRD, ICP-MS
S-B-Coating	Coatings abraded off black part of stratified type	XRD, ICP-MS
N-Y-Coating	Coatings abraded off yellowish-red part of nodule-cemented concretion type	XRD, ICP-MS
N-B-Coating	Coatings abraded off black part of nodule-cemented concretion type	XRD, ICP-MS
BR	Beach rock	ICP-MS
BIOS	Bacteriogenic iron oxides	ICP-MS
BM	Beach mud	ICP-MS
RS	Red soil from weathering beach rock	ICP-MS
WS	White soil from weathering beach rock	ICP-MS
SW	Seawater	ICP-MS
GW	Groundwater	ICP-MS

### 3.3 Electron microscopy

#### (1) Backscattered electron (BSE) image—X-ray mapping analysis

BSE images of N-B and N-Y samples were obtained in the BSE mode of the Electron Microprobe Analyzer (EMPA) JXA-8100 (JEOL) after carbon coating on the thin sections at Institute of Geology and Geophysics, Chinese Academy of Sciences (CAS). The element distributions of the selected area of N-B and N-Y were obtained by X-ray mapping using a link INCA (Oxford) microanalytical energy dispersive X-ray spectrometer (EDS) system of the JXA-8100 (JEOL).

#### (2) Scanning electron microscopy (SEM)—energy dispersive X-ray spectrometer (EDS) analysis

The original samples (S-Y, S-B, N-Y, N-B) were gold-coated and their surface features were examined by SEM (Sirion 200) using accelerating voltages of 20 kV or 5 kV. A Link INCA microanalytical EDS system was used to determine the spot elemental compositions of the samples. Environmental Scanning Electron Microscopy (ESEM) (XL 30) was used to observe the gold-coated section of S-type with an accelerated voltage of 15 kV, and the element distributions of the selected area were obtained by X-ray mapping using a link INCA microanalytical EDS system.

### 3.4 X-ray powder diffraction (XRD)

Powder X-ray diffraction was used to determine the mineralogy and crystallinity of the subsamples (S-Y-Coating, S-B-Coating, N-Y-Coating, N-B-Coating) by D/MAX-rA diffractometer (Rigaku, Japan) (operating at 40 kV and 40 mA, Cu K*α* radiation), with scanning range from 10° to 70°, a step size of 0.02°, and a rate of 0.5 s/step. Then, Jade 6.5 analytical software was used to assign the collected peaks.

### 3.5 Inductively coupled plasma—mass spectrometry (ICP-MS)

Trace elements of the subsamples (S-Y-Coating, S-B-Coating, N-Y-Coating, N-B-Coating) and related environmental materials (BR, BIOS, RS, WS, BM) were measured by ICP-MS (Perkin-Elmer Elan DRC II). Approximately 100 mg dried powder samples were placed in pre-cleaned Teflon-lined microwave digestion bombs, followed by 6 mL of ultrapure HNO_3_ and 3 mL of ultrapure HF. The sealed digestion bombs were heated at 140 ^o^C for 5 min, and then 185 ^o^C for 15 min in a microwave oven (CEM-MARS-X-press). After cooling down, each digested sample was mixed with 0.2 mL HClO_4_. The samples were subsequently heated again with electric heating under 180^o^C to be almost dry (about 1 mL), followed by adding 6 mL ultrapure HNO_3_. Then the digestion bombs were sealed and heated at 150^o^C for 1–2 hrs. After cooling, the dissolved samples were decanted into clean polyethylene bottles and diluted by a factor of 1000 before analysis by ICP-MS. The seawater (SW) and the groundwater (GW) samples were first filtered through 0.20 μm films, then 5× pre-concentrated on electric heating at 50 ^o^C, and again filtered via 0.20 μm films after cooling before analysis by ICP-MS. ^103^Rh was used as an internal standard and the US Geological Survey standards BHVO-2, BIR-1 and GSP-2 were used as external standards for quality control. The analytical errors were generally less than 5%.

## Results

### 4.1 Micrographs of thin sections

Thin sections of S-Y, S-B, NY, N-B samples were examined by plane polarized light (Olympus-BX51-P) (Fig. 2-S-Y(1), S-B(1), N-Y(1), N-B(1)). These samples were mainly composed of sand grains, such as quartz and feldspar, which were weathering products of beach rocks. The sand grains in the S-Y and S-B samples were subangular and about 100 μm long. In contrast, the sand grains in the N-Y and N-B samples were more round and about 60–80 μm long. The sand grains in S-Y and N-Y were coated by yellowish-red materials, and in S-B and N-B by black materials. In S-Y, the sand grains were immersed into the yellowish-red materials and had a more than 30 μm thick coating (Fig. 2-S-Y(1)). The coating in S-B was much thinner at 10–20 μm (Fig. 2-S-B(1)). The magnified SEM images showed that these coatings were very coarse and loose, and attached on the surface of grains (Fig. 2-S-Y (2),-S-B-(2)). The coatings in N-B (20–50 μm) were thicker and more dense than those in N-Y (10–20 μm) (Fig. 2-N-Y(1),-N-B-(1)). The BSE signal was strongly dependent on the mean atomic number of target constituents [33], so the present BSE images showed that the coatings had larger atomic numbers than the sand grains. The coatings were shaped like crenulated crystals around the sand grains in N-Y (Fig. 2-N-Y(2)) and netty cementation between the sand grains in N-B (Fig. 2-N-B(2)).

### 4.2 X-ray mapping

**Fig 3:**
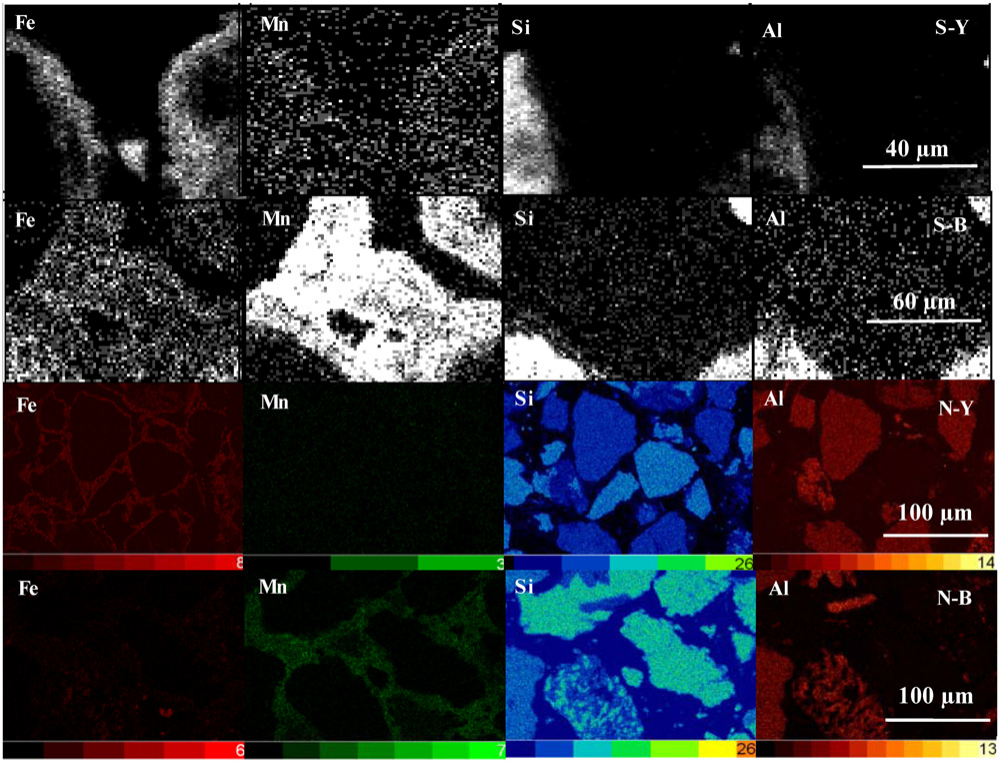
Digital mapping of the different precipitates among the sand particles in two typical samples. S-type samples from the pink squares in Fig. 2 S-Y (2) and S-B (2) were carried on Environmental Scanning Electron Microscopy (ESEM)-X-Ray mapping (top two), and N-type samples from Fig. 2 N-Y (2) and N-B (2) were performed on Electron Microprobe Analyzer (EMPA)-X-Ray mapping (bottom two).

To find out the element distribution patterns in the samples, we checked selected areas of the S-Y, S-B, N-Y and N-B samples by X-ray mapping. The quartz grains were encrusted by Fe minerals plus minor Mn minerals in S-Y, and by Mn minerals plus minor Fe minerals in S-B. The distributions of Fe in S-Y and Mn in S-B appear very uniform (Fig. 3S-Y, S-B). In N-Y and N-B, the randomly oriented quartz grains were cemented in Fe or Mn matrix for yellowish-red part and black part, respectively. The distributions of Fe or Mn in N-Y or N-B were also very uniform (Fig. 3N-Y, N-B).

### 4.3 X-ray diffraction

**Fig 4:**
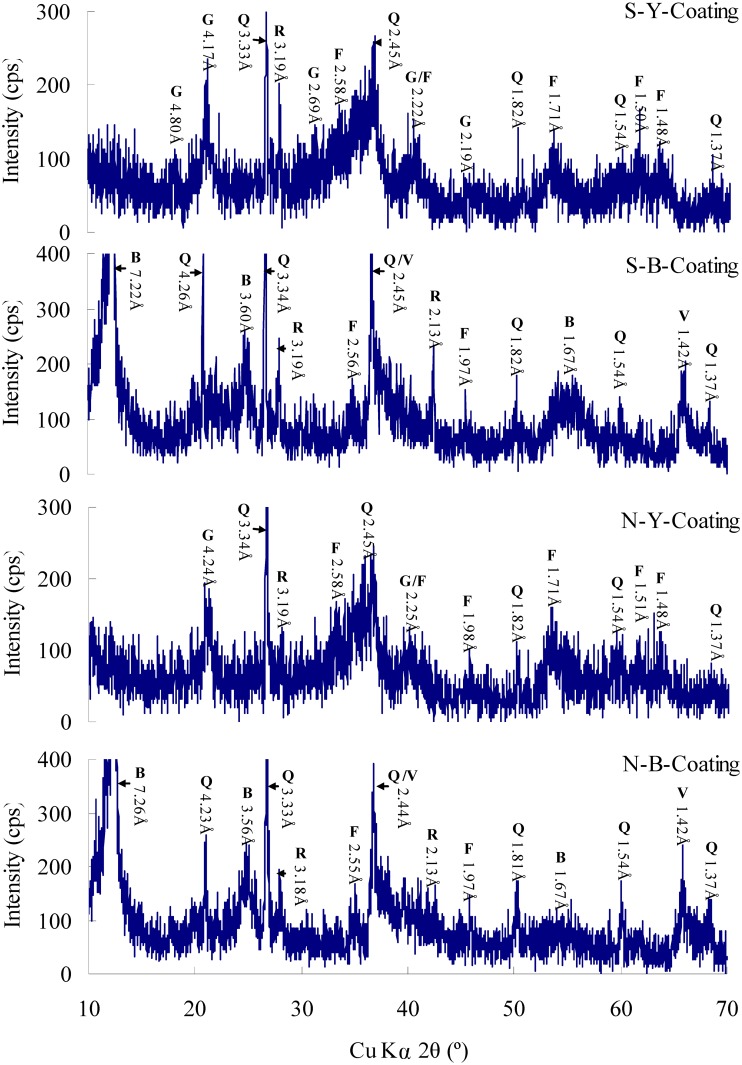
XRD patterns of different coatings in two typical samples. Y-Coating: Q-Quartz; R-Rutile; F-Ferrihydrite; G-Goethite. B-Coating: Q-Quartz; R-Rutile; F-Ferrihydrite; B-Birnessite.

The XRD powder analysis showed that the yellowish-red coatings had very similar mineral compositions and mainly consisted of quartz (Q), rutile (R), ferrihydrite (F), and goethite (G), in which ferrihydrite was characterized by some small and very broad reflections with d-spacing of 2.58, 2.25, 1.71, 1.98, 1.51, 1.48 Å in S-Y-Coatings and 2.58, 2.25, 1.98, 1.71, 1.51, 1.48 Å in N-Y-Coatings, and goethite was characterized by small and broad peaks corresponding to d-spacings of 4.80, 4.17, 2.69, 2.22 and 2.19 Å in S-Y-Coatings and 4.24 and 2.25 Å in N-Y-Coatings (Fig. 4-S-Y-Coating, N-Y-Coating). The major minerals in the black coatings were identified as quartz (Q), rutile (R), birnessite (B) and ferrihydrite (F) by XRD patterns, in which birnessite was characterized with d-spacings of 7.22, 3.60, 3.19, 2.56, 2.45, 1.67, 1.42 Å in S-B-Coatings and 7.26, 3.56, 2.55, 2.44, 1.67, 1.42 Å in N-B-Coatings (Fig. 4-S-B-Coating, N-B-Coating). The major d values of these natural Fe and Mn oxides minerals were compared to synthetic ferrihydrite (data from JCPDS card 29–0712), goethite (data from JCPDS card 81–0464), and birnessite (data from JCPDS card 43–1456), respectively. Therefore, the metal minerals in the yellowish-red coatings were the yellowish hydrous ferric oxides (HFO), and those in the black coatings were black manganese oxyhydroxides plus minor iron oxyhydroxides. Furthermore, the low intensity and the great breadth and asymmetry of XRD patterns indicated high degree of disorder, small particle size, and low crystallinity of these Fe/Mn oxides [12].

### 4.4 Micromorphological features and EDS microprobe analysis

**Fig 5:**
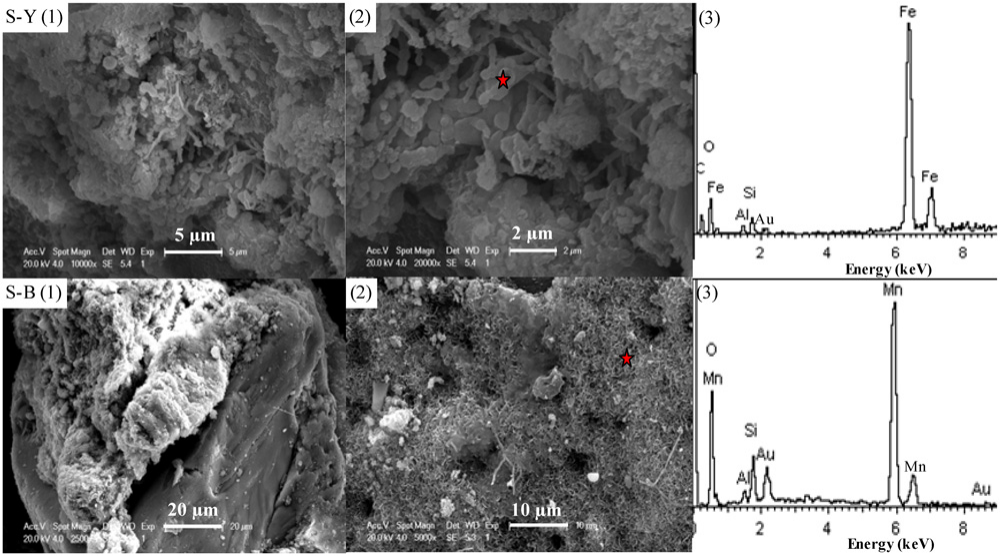
Scanning electron microscopy (SEM) photos of S-type sample and Energy diffraction spectrum (EDS) of selected areas (marked with red star) in SEM images. The top showed the images/spectrum of S-Y part, and the lower displayed the feature of S-B part.

SEM observations showed a large number of poorly crystalline spheres with diameters less than 1μm and globular aggregations on the surface of S-Y and communities of 2 μm long filamentous objects in the cavities (Fig. 5S-Y (1)). On closer examination, the filamentous objects were composed of cocci-like units (Fig. 5S-Y (2)) characterized by Fe, plus minor Si and Al, as identified by EDS analysis (Fig. 5S-Y (3)). Similar filamentous units were found in the banded Fe-Mn precipitates in Akayu hot spring and Yuno-Taki, Japan [36–37]. Combined with XRD results, the spheres and globular aggregations in the SEM images were identified as ferrihydrite minerals [14–15].

In contrast, about 20 μm thick coarse materials, attached on the surface of quartz particles, were observed on the surface of S-B (Fig. 5S-B (1)). A magnification of the coarse materials showed that the coatings consisted of numerous tiny crenulated platy crystals arranged in irregular boxwork structures (Fig. 5S-B (2)). These crenulated platy crystals clusters were composed of Mn plus minor Si and Al (Fig. 5S-B (3)). Combined with their characteristics of morphology, these crystals were identified as birnessite [11, 27, 38–39]. Some filamentous objects, more than 10 μm long, co-existed with the birnessite mineral clusters (Fig. 5S-B (2)).

**Fig 6:**
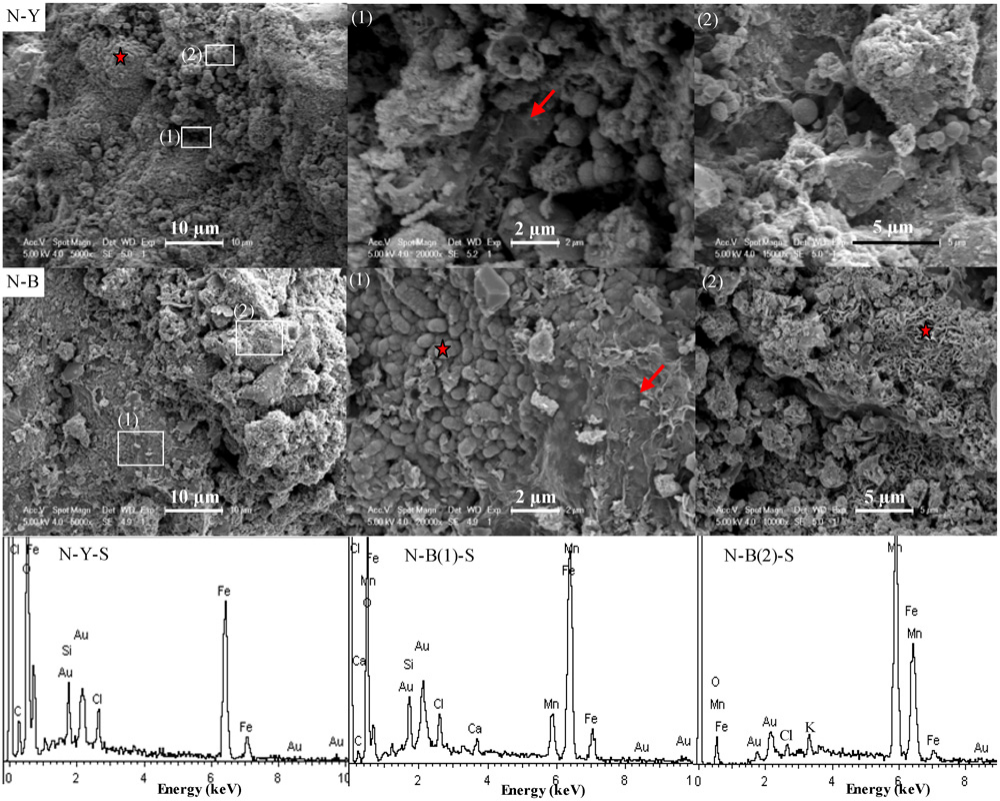
Scanning electron microscopy (SEM) photos of N-type sample and Energy diffraction spectrum (EDS) of selected areas (marked with red star) in SEM images. The top showed the SEM images of N-Y part, the middle displayed the feature of N-B, and the bottom were the spectrum of EDS. The red arrows indicated the biofilm-like object/remnants there.

Numerous spheres with diameters of about 1 μm and their clusters occurred on the surface of N-Y (Fig. 6N-Y) and these spheres were composed of Fe plus minor Si and Cl, as identified by EDS analysis (Fig. 6N-Y-S). The magnifications on selected areas in Fig. 5N-Y showed that the biofilms-like object with an area of 2 μm × 4 μm in vision was under the sphere clusters (Fig. 6N-Y (1)) and the spirillum-like object, about 3 μm long, was connected to the spheres (Fig. 6N-Y (2)). The spirillum-like object was very similar to the helical stalks of *Gallionella ferruginea* (G) in shape [40]. On the surface of N-B, subsphere particles with diameter of less than 1 μm long were clustered together and associated with biofilm-like remnants (Fig. 6 N-B (1)). The crenulated platy crystal clusters in Fig. 6 S-B were also extensively present in N-B (Fig. 6 N-B (2)), and covered subsphere particles there (Fig. 6 N-B). The EDS analysis likely indicated the subsphere particles as birnessite ((Na, Ca)Mn_7_O_14_.2.8H_2_O), which contained high contents of Mn plus minor Ca in Fig. 6 N-B(1)-S, and K instead of Na in Fig. 6 N-B(2)-S. The observed minor Fe, Si and Cl were likely adsorbed onto or incorporated into these biogenic Mn oxides [7, 9, 12, 41–42].

### 4.5 Middle rare earth element (MREE) enrichments in ferromanganese coatings

The REE elements were divided into three groups: (1) LREE, including La, Ce, Pr, Nd; (2) MREE, including Sm, Eu, Gd, Tb, Dy, Y, Ho; (3) HREE: Er, Tm, Yb, Lu. The concentrations of REEs in the coatings and other related materials were listed in S1 Table. Concentrations of ∑REE in the coatings ranged from 220–270 ppm, a little higher than those of RS, WS and BM. In contrast, BIOS of the seepage water in the intertidal zone had only about 100 ppm of ∑REE, much lower than those of the coatings, but similar to that of BR. Concentrations of ∑REE in SW and GW were 715 and 382 ppt, respectively (Table 2).

**Table 2:** Summary statistics for REE in the coatings and related materials in the intertidal area, East China Sea.

	BR	RS	WS	BIOS	BM	S-Y-Coating	S-B-Coating	N-Y-Coating	N-B-Coating	SW	GW
∑REE (ppm)	106.93	205.77	205.47	107.20	233.22	269.39	234.64	226.92	268.88	0.72	0.38
[La/Pr]_PAAS_	1.26	1.10	1.12	1.04	1.01	0.97	1.00	0.93	0.91	1.76	1.35
[La/Sm]_PAAS_	1.62	1.11	1.18	0.92	0.88	0.80	0.85	0.74	0.71	1.20	1.45
[La/Yb]_PAAS_	0.89	1.16	1.46	1.62	1.24	1.32	1.43	1.45	1.32	1.65	1.65
[Gd/Dy]_PAAS_	0.95	1.25	1.39	1.46	1.20	1.21	1.15	1.25	1.18	1.04	1.29
[Gd/Yb]_PAAS_	0.61	1.11	1.26	1.88	1.51	1.87	1.83	2.18	2.02	1.21	1.61
[Er/Yb]_PAAS_	0.76	0.82	0.84	1.06	1.03	1.11	1.14	1.15	1.15	3.88	7.17
[Y/Ho]_PAAS_	1.26	1.08	1.31	1.03	1.03	0.84	0.85	0.90	1.02	2.26	0.98
Ce/Ce*	1.15	1.38	1.33	0.87	1.13	1.52	1.04	0.62	0.78	2.44	1.79
Eu/Eu*	0.63	0.75	0.76	0.83	0.93	0.92	0.94	0.91	0.94	1.25	0.64

In order to assess the variations in REE patterns between samples, we normalized the measured REEs to Post-Archean Australian Shale (PAAS) (La 38.2, Ce 79.6, Pr 8.83, Nd 33.09, Sm 5.55, Eu 1.08, Gd 4.66, Tb 0.774, Dy 4.68, Y 27, Ho 0.991, Er 2.85, Tm 0.405, Yb 2.82, Lu 0.433 ppm) [43] (Table 2). [La/Pr]_PAAS_, [Gd/Dy] _PAAS_ and [Er/Yb] _PAAS_ were employed to represent the fractionations among LREE, MREE and HREE, respectively. [La/Sm] _PAAS_ was used to distinguish between LREE and MREE, [La/Yb] _PAAS_ between LREE and HREE, and [Gd/Yb] _PAAS_ between MREE and HREE. PAAS-normalized Y anomaly was expressed as [Y/Ho] _PAAS_, Ce anomaly as Ce/Ce* = Ce_PAAS_/(La_PAAS_*Pr_PAAS_)^0.5^, and Eu anomaly as Eu/Eu* = Eu_PAAS_/(Sm_PAAS_*Gd_PAAS_)^0.5^, where PAAS indicated PAAS-normalized values [44].

**Fig 7:**
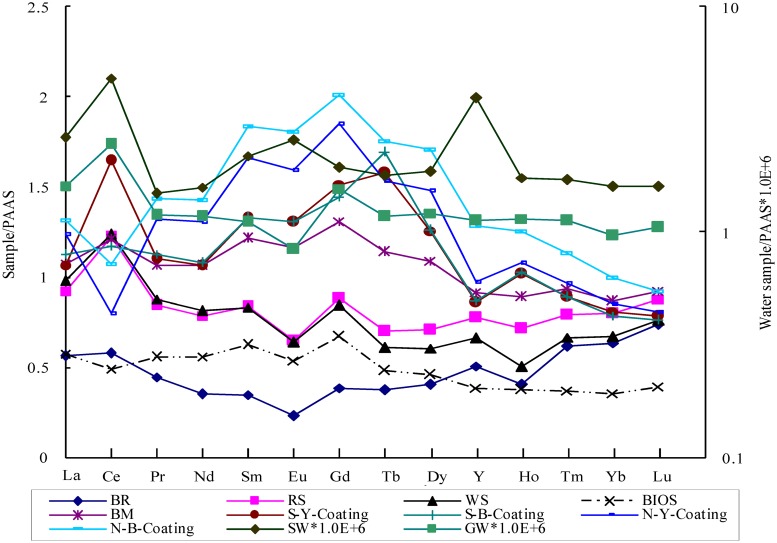
REE patterns normalized by PAAS. BR, RS and WS were characterized by middle REE depletion. However, BIOS, BM and ferromanganese coatings were characterized by a distinct middle REE hump. The normalized pattern of GW was almost flat, and SW with small MREE enrichment.

Overall, the PAAS-normalized REE patterns of the coatings displayed apparent MREE enrichment with minor negative Eu anomalies as revealed by [La/Sm]_PAAS_ (0.71–0.85), [La/Yb]_PAAS_ (1.32–1.45), [Gd/Yb]_PAAS_ (1.83–2.12), and [Eu/Eu*]_PAAS_ (0.91–0.94). There existed the positive Ce anomalies for the S-Y-Coating ([Ce/Ce*]_PAAS_ = 1.52), negative Ce anomalies for the N-Y-Coating ([Ce/Ce*]_PAAS_ = 0.62) and N-B-Coating ([Ce/Ce*]_PAAS_ = 0.78), and no Ce anomalies for the S-B-Coating ([Ce/Ce*]_PAAS_ = 1.04). There also existed positive Tb anomalies in the S-Y-Coating and the S-B-Coating, but no Tb anomalies in N-type samples. Similar PAAS-normalized REE patterns were also found in the BIOS and BM samples ([La/Sm]_PAAS_ (0.88–0.92), [La/Yb]_PAAS_ (1.24–1.62), [Gd/Yb]_PAAS_ (1.51–1.88), and [Eu/Eu*]_PAAS_ (0.83–0.93)) with positive Ce anomalies in BM ([Ce/Ce*]_PAAS_ = 1.13), negative Ce anomalies in BIOS ([Ce/Ce*]_PAAS_ = 0.87), and no Tb anomalies in either BM and BIOS. MREE was depleted in BR as judged by [La/Sm]_PAAS_, [La/Yb]_PAAS_, and [Gd/Yb]_PAAS_ ratios of 1.62, 0.89, and 0.61, respectively. Minor positive Ce and Y anomalies and negative Eu anomalies in BR were identified by [Ce/Ce*]_PAAS_, [Y/Ho] _PAAS_, and [Eu/Eu*] _PAAS_ ratios of 1.15, 1.26, and 0.63, respectively. The weathering products of BR (RS and WS) had very similar PAAS-normalized REE patterns with small LREE enrichment ([La/Sm]_PAAS_ (1.11–1.18), [La/Yb]_PAAS_ (1.16–1.46), and [Gd/Yb]_PAAS_ (1.11–1.26)), positive Ce anomalies ([Ce/Ce*]_PAAS_ (1.33–1.38)), and negative Eu anomalies ([Eu/Eu*]_PAAS_ (0.75–0.76)). For GW, the PAAS normalized REE pattern was almost flat except positive Ce and Er anomalies, negative Eu anomalies, and a higher La value. Small MREE enrichment was observed in SW with positive Eu and Y anomalies (Table 2 and Fig. 7).

### 4.6 Trace metal partitioning in ferromanganese coatings

**Fig 8:**
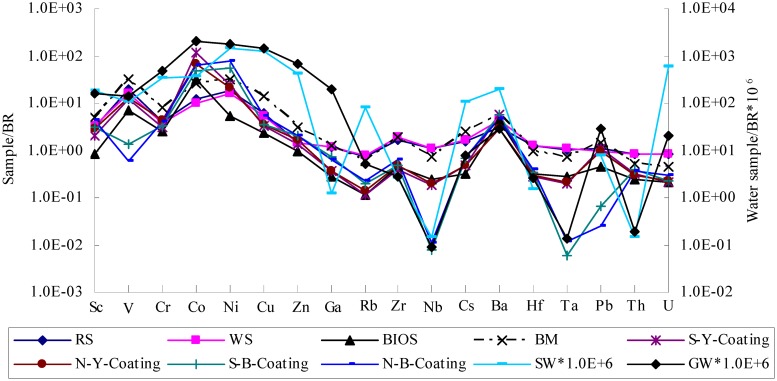
Trace metal elements in the coatings and related materials were normalized by BR.

The BR-normalized trace metal patterns of the samples displayed a similar distribution with enrichment in Sc, V, Cr, Co, Ni, Cu, Zn, and Ba, and depletion in Ga, Rb, Zr, Nb, Cs, Hf, Ta, Pb, Th, and U (Fig. 8). In the yellowish-red coatings (S-Y-Coating and N-Y-Coating), Co had the highest enrichment factor (EF) of 122 and 66.6, respectively. V and Ni exhibited moderate enrichment (EF = 10–20), and the Sc, Cr, Cu and Ba had low enrichment (EF = 2–6). Zn and Pb had EF values close to 1. Other elements were depleted with EF <0.5. In the black coatings (S-B-Coating and N-B-Coating), Co and Ni had very high EF factors (50–70), and Sc, Cr, Cu, Zn and Ba had very low EF factors (2–6). Nb, Ta and Pb were very depleted (EF = 0.01–0.06). Other elements were moderately depleted (EF = 0.2–0.7). In the weathering profiles of WS and RS, trace elements were enriched to varying extents as indicated by very high EF factors for V, Co, Ni (10–19), moderate values for Sc, Cr, Cu, Ba (3–6), and close to or less than 1 for other elements. The BIOS was characterized by strong adsorption of Co (EF = 32) and moderate adsorption of V, Cr, Ni, Cu, and Ba (EF = 2.5–7). Different from BIOS, the BM was strongly enriched by V (EF = 33), Co (EF = 25.5), Ni (EF = 31), and Cu (EF = 13.7), and moderately by Sc (EF = 5), Cr (EF = 8), Zn (EF = 3), Zr (EF = 1.85), Cs (EF = 2.62), and Ba (EF = 5.78). In the water samples, their BR-normalized trace metal patterns were similar to those of the coatings, characterized by strong enrichment of Cr, Co, NI, Cu, Zn, Sc, and V. Moreover, GW also had high contents of U, Rb, Cs and Ba, and SW had high contents of Ga.

## Discussion

### 5.1 Structure of bacteriogenic ferromanganese coatings and their scavenging properties on trace metal elements

High degrees of disordered ferrihydrite, goethite and birnessite were usually associated with bacteria in natural environment, and could be used as biogenic indicators [7–8, 13]. Typical morphologies of biogenic Fe or Mn oxides were nanocrystalline, fibrous aggregates resembling crumples or sphere structures [45–47]. In the present study, the observed fossilized bacterium-like and biofilm-like remnants suggested that bacteria likely played an important role in the formation of the Fe-Mn coatings. Similar birnessite boxwork aggregates were described by Melim et al. (2008), Jones (2009) and Miller et al. (2012) [12, 48–49]. Microorganisms were known under certain conditions to accelerate the rate of Fe-Mn oxidation up to several orders of magnitude as compared to abiotic oxidation [7, 13, 50]. Recently, Learman et al.(2011) [51] revealed that Mn(II) could be oxidized indirectly by bacteria via the enzymatic generation of extracellular superoxide radicals.

**Fig 9:**
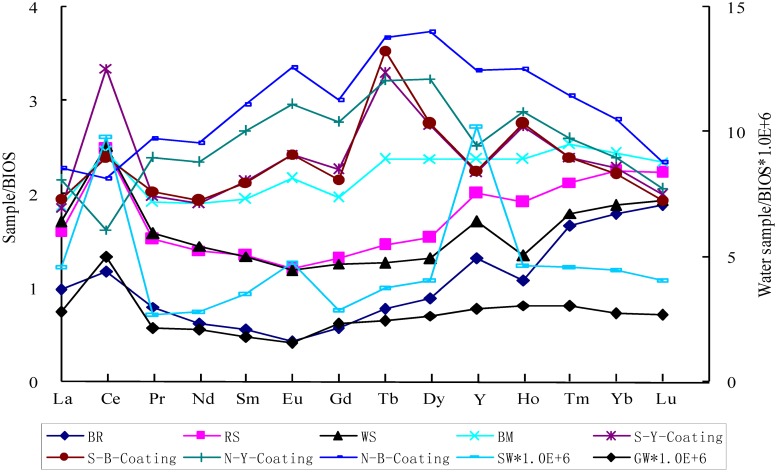
REE patterns normalized by BIOS. BR, RS, WS and BM were characterized by middle REE depletion. However, SW and ferromanganese coatings were characterized by a distinct middle REE hump. The normalized patterns of GW were almost flat.

Furthermore, enrichment of heavy metals and alkaline earth elements (Co, Ni, Sc, Cr, Cu, Ba) corroborated our conclusion that ferromanganese coatings were trace metal element scavenger [7, 52]. Two factors likely attributed to the metal adsorption/co-precipitation by the ferromanganese coatings: (1) the physico-chemical properties of the mixing solution from the groundwater and the seawater in the intertidal zone and of the seawater after the flocculation; (2) the modality of growth and the structural effect on adsorption/co-precipitation [38]. As for the first factor, the principle properties affecting adsorption/co-precipitation of trace metals included pH, the nature of complexing agents, and the ionic strength of the mixing water. In the present study, pH of the mixing water was 6.00 that should favor trace metals adsorption/co-precipitation onto Fe-Mn oxyhydroxides. In SW, the major anions were SO_4_^2-^ and Cl^-^, and the major cations were K^+^, Na^+^, Mg^2+^, and Ca^2+^ [33]. Most of Cu, Pb, and U (60–100%) and small amount of Ba, Co, Ni, and Zn (10–20%) would bind to SO_4_^2-^, and some of Pb and Cd (40%) and small amount of Co, Ni, Zn, and Cu (10–20%) would form complexes with Cl^-^. Rb, Cs, Ba Co, Ni, and Zn were mainly present as free cations, but Sc, Zr, Nb, Ta, and Th as hydroxide complexes. V and Cr were present as oxyanions [53]. Co, Ni, Zn, Ba were adsorbed preferentially on the negative charged surface of Mn-oxyhydroxides, and all sulfate complexes (Cu, Pb) and hydroxide complexes (Sc) and oxyanions (V, Cr) bind to the slightly positively charged surface of the amorphous Fe-oxyhydroxides [53]. Moreover, GW and SW were also enriched in Sc, V, Cr, Co, Ni, Cu, and Zn as compared with the other elements (Fig. 9). Therefore, Sc, V, Cr, Co, Ni, Cu, Zn, and Ba were strongly enriched in the coatings.

Regarding for the second factor, the particle size of Fe-Mn oxyhydroxides strongly influenced the adsorption capacity with greater adsorption observed on smaller crystals [38, 54]. In the present study, the Fe-Mn coatings could strongly scavenge heavy metals from environmental solutions. Recent studies showed that the biogenic iron/manganese iron oxides were dominant sorbents for heavy metals in aquatic environment because of larger surface area, high reactivity, and partly degraded bacterial cells [7, 55–56]. Even a rather rapid accretion rate of coatings or nodules could in turn affect the extent of adsorption/co-precipitation [38].

### 5.2 Origin of MREE and formation mechanism

To trace the formation processes of coatings, BIOS-normalized REE patterns were employed, which were expressed as REE_sample_/REE_BIOS_ (Fig. 9). The BIOS-normalized REE patterns of BR, RS and WS exhibited apparent MREE depletion, and the patterns of SW and GW were almost flat except a few anomalies. However, BM and coatings had apparent MREE enrichment patterns. Moreover, the BIOS-normalized patterns between different parts of S-type samples almost overlapped with minor positive Ce anomalies, negative Gd and Y anomalies, and strong Tb anomalies. Only the negative Ce anomalies and minor positive Tb anomalies were observed in the N-type samples.

In the present study site, the ancient wood layers could supply abundant humic substances (HS) via fermentation [33]. HS could act as electron donor and electron shuttle for bacteria to increase the Fe-Mn reducing rate [57–60]. Furthermore, previous studies revealed that iron-oxidizing bacteria [34–35] and sulfate-reducing bacteria [33] were extensively present in the present study site. The HS-rich organic colloid solutions mixed with groundwater and flowed out to form the seepage water systems, which contained high-content organic matters, and active iron bacteria and sulfate-reducing bacteria [33–35]. The Fe-Mn minerals likely existed in colloid or solution form [28, 61–62]. Compared with HFO or MnO_2_, REE(III) were preferentially bounded to HS to form HS-REE complexes even at low concentrations of HS, and this binding slowed down the adsorption of REE(III) onto the surface of HFO or MnO_2_ [62–65]. Positive Ce anomalies and well-developed tetrad effect were observed in HFO or MnO_2_ suspensions, but not in HS solution [63–65]. Thus, minor MREE enrichments and few Ce anomalies were observed on the BIOS precipitation from the groundwater. The corresponding groundwater was also characterized as almost flat distribution patterns with exception at La, Ce and Er, which was likely caused by the intrusion of SW.

The REE fractionations could occur during the mixing of the acidic (pH = 2.60) groundwater and the circum-neutral pH (6.00) seawater. In this process, the REE fractionations were controlled by (1) pH: With increasing pH, REEs were absorbed onto particle surface in the order of LREEs>MREEs>HREEs, and with decreasing pH, REEs were released from surfaces in the same order; (2) Coagulation: The coagulation took place in the mixing zone, followed by fractionation with the order of LREEs>MREEs>HREEs; (3) Salinity: As the salinity of mixing solution from low (S = 0–5), middle (S = 5–9) to high (S = 9–35), the REEs experienced a removal by a reduction of 40–50% with the exception of La and Y which reduced to approximately 60% of the initial abundance, re-mineralization by a strong increase to 60–100% with the exception of La and Y which increase to 200% and 130% of the initial abundance, respectively, and a rapid drop by 15–30% with the exception of Y which sharply drop to 65% at a salinity of 17, then gradually increase to 130% at a salinity of 35 [66]. In the present study, the salinity of SW was high at 26.9. Thus, the mixing processes should be dominated by the removal from the dissolved and solution pool. The rapid removal of REEs from the pool might reach 60–80% of total amount of REEs and followed the order of LREE>MREE>HREE [61, 66]. Furthermore, the removal processes could result in strong La, Y, Er anomalies in SW according to Lawrence & Kamber (2006) [66]. The positive Ce anomalies in SW could be caused by the weak Ce capture and slow oxidization on particles or ferromanganese oxides (Eh of-612.8 mV) because of the existence of ancient wood layers [33].

Based on the controlling factors of mixing processes, the Fe-Mn coatings in the present study should have a shale-normalized REEs pattern with LREE>MREE>HREE, and the corresponding solution should be characterized by LREE<MREE<HREE. However, the coatings and BM in the present study displayed strong MREE enrichment patterns. Thus REEs fractionation likely happened during the flocculation of hydrous Fe-Mn oxides after precipitation. Similar MREE enrichment patterns were reported in the previous works because of precipitation of iron oxyhydroxide in natural environment [67–70].

Scavenging experiments performed by Bau (1999) [71] showed that at pH ≥5, the patterns of REE distributions on iron-oxyhydroxide displayed the M-type lanthanide tetrad effect and anomalous behavior of La, Gd, and possibly Lu with negative anomalies. Many researches reported that Fe-Mn oxides/oxyhydroxide were commonly enriched in the MREEs with respect to shales [62]. Johannesson et al. (1996) [72] suggested that MREE-enrichment in acidic lake and groundwaters was due to dissolution of Mn- and Fe- oxyhydroxides in the aquifer materials.

The differences between S-type sample and N-type sample in Ce anomalies were caused by the fact that the S-type sample was formed near the high tide level and most of time exposed to air. Thus, the Ce(III) was readily oxidized into CeO_2_. The iron-oxyhydroxide was formed before manganese-oxyhydroxide because of high Eh-pH region for manganese-oxyhydroxide, thus the Ce(III) was preferentially oxidized and adsorbed onto the surface of iron-oxyhydroxide, causing stronger positive Ce anomalies than that of Mn-oxyhydroxide. In contrast, the N-type sample was formed near low tide level and dominated by low pH (pH = 6.0), weak Ce capture and slow oxidization rate (Eh = -612.8 mV) in the intertidal seawate [33]. In this case, the REE patterns of N-type sample were characterized by negative Ce anomalies. However, Ce oxidation typically occurred due to surface catalysis on Mn hydroxides [71, 73], but oxidative scavenging of Ce on Fe oxyhydroxides had not been observed yet [74–75]. Therefore, the N-B-Coating mainly composed of Mn-oxyhydroxides had smaller positive Ce anomalies than that of the N-Y-Coating, which was mainly composed of Fe-oxyhydroxides. For the positive Tb anomalies in S-type sample, not in N-type sample, we had no good explanations.

## Conclusions

In an intertidal zone influenced by ancient wood layers in East China Sea, extensive ferromanganese coatings on sand grains were discovered. The main mineral compositions of coatings were ferrihydrite and goethite for the yellowish-red parts, and birnessite for the black parts. SEM observations show that there were lots of poorly crystalline spheres and globular aggregations, co-existing with communities of filamentous or biofilm-like remains on the surface of yellowish-red coatings. Meanwhile, numerous tiny crenulated platy crystals assembled as irregular boxwork structures on the surface of black coatings. Moreover, the bacteria-like remnants were also observed in the black coatings. Thus, it was very likely that bacteria played an important role in the formation of Fe-Mn oxyhydroxide coatings here. The chemical results indicated that trace metals of the coatings were enriched with Sc, V, Cr, Co, Ni, Cu, Zn, Ba, particularly for Co, Ni. PAAS-normalized MREE enrichment patterns of the coatings were caused by two subsequential processes: (1) preferentially release of Fe-Mn from the beach rocks by fermentation of ancient woods and colloidal flocculation in the mixing water zone; (2) preferentially adsorption of MREE by Fe-Mn oxyhydroxides from the seawater. These findings in the present study provided a sight in the microscale features of Fe-Mn oxyhydroxide coatings and the Fe-Mn biogeochemical cycles involved buried organic matters in the intertidal zone or shallow coast.

## Supporting Information

S1 Table Trace elements concentrations of different materials in intertidal zone by ICP-MS. (DOC)
