# Correction: Microscale Characterization and Trace Element Distribution in Bacteriogenic Ferromanganese Coatings on Sand Grains from an Intertidal Zone of the East China Sea

**DOI:** 10.1371/journal.pone.0126301

**Published:** 2015-04-13

**Authors:** The PLOS ONE Staff

There are errors in the Funding section. The correct funding information is as follows:

This work was supported by National Natural Science Foundation of China (NNSFC31400091) (http://www.nsfc.gov.cn), Natural Science Foundation of Jiangsu Province (BK2012202) (http://www.jstd.gov.cn/), Chinese Postdoctoral Science Foundation (2013M540518) (http://res.chinapostdoctor.org.cn/BshWeb/index.shtml), Jiangsu Provincial Postdoctoral Science Foundation (1302080C) (http://www.jsbsh.gov.cn/), National 973 Program (Grant No 2010CB428902) (http://www.most.gov.cn/), and Applied Basic Research Project of Suzhou (SYN201306) (http://www.szkj.gov.cn/default.html). The funders had no role in study design, data collection and analysis, decision to publish, or preparation of the manuscript.

The publisher apologizes for the error.
