# Supplementary material for: Microscale Characterization and Trace Element Distribution in Bacteriogenic Ferromanganese Coatings on Sand Grains from an Intertidal Zone of the East China Sea
Source: PLoS One. 2015 Mar 18;10(3):e0119080. doi: 10.1371/journal.pone.0119080 (PMC4365015; doi:10.1371/journal.pone.0119080)
Supplement: S1 Table — (DOC) [file pone.0119080.s001.doc]

**Supporting Information**

**Table S1. Trace elements concentrations of different materials** in intertidal zone by ICP-MS.

| Element | Atomic number | BR | RS | WS | BIOS | BM | S-Y  -Coating | N-Y  -Coating | S-B  -Coating | N-B  -Coating | SW | GW |
| --- | --- | --- | --- | --- | --- | --- | --- | --- | --- | --- | --- | --- |
|  |  | ppm | ppm | ppm | ppm | ppm | ppm | ppm | ppm | ppm | ppt | ppt |
| Sc | 45 | 3.37 | 10.20 | 11.10 | 2.76 | 16.60 | 6.97 | 9.63 | 10.20 | 15.20 | 644 | 545 |
| V | 51 | 3.82 | 74.50 | 63.60 | 27.60 | 125.00 | 45.90 | 51.80 | 5.32 | 2.31 | 398 | 557 |
| Cr | 53 | 11.60 | 47.50 | 45.70 | 29.60 | 93.30 | 35.00 | 51.10 | 38.80 | 47.70 | 3945 | 5636 |
| Co | 59 | 0.68 | 8.23 | 6.76 | 21.80 | 17.40 | 83.20 | 45.30 | 33.30 | 43.90 | 255 | 1364 |
| Ni | 60 | 1.42 | 25.70 | 22.60 | 7.56 | 44.50 | 35.70 | 29.90 | 76.70 | 108 | 2107 | 2568 |
| Cu | 65 | 3.10 | 19.70 | 17.20 | 7.48 | 42.50 | 10.00 | 10.90 | 11.30 | 16.80 | 3945 | 4523 |
| Zn | 66 | 32.30 | 48.70 | 47.10 | 31.80 | 102.00 | 44.10 | 54.70 | 64.70 | 68.40 | 13564 | 22386 |
| Ga | 71 | 17.60 | 20.80 | 20.20 | 4.92 | 21.90 | 6.44 | 6.46 | 12.90 | 11.50 | 21 | 3545 |
| Rb | 85 | 295.00 | 222.00 | 228.00 | 34.20 | 180.00 | 34.10 | 42.00 | 57.10 | 68.30 | 24429 | 1589 |
| Y | 89 | 13.70 | 20.90 | 17.80 | 10.40 | 24.70 | 23.20 | 26.30 | 23.50 | 34.60 | 107 | 30 |
| Zr | 90 | 84.00 | 145.00 | 160.00 | 38.30 | 156.00 | 33.10 | 40.10 | 40.40 | 54.00 | 221 | 236 |
| Nb | 93 | 23.20 | 25.10 | 25.10 | 5.55 | 16.50 | 4.20 | 4.51 | 0.18 | 0.26 | 3 | 2 |
| Cs | 133 | 4.28 | 6.71 | 7.28 | 1.42 | 11.20 | 2.06 | 2.04 | 3.02 | 3.14 | 471 | 34 |
| Ba | 135 | 70.70 | 225.00 | 303.00 | 303.00 | 409.00 | 411.00 | 202.00 | 414.00 | 323.00 | 14048 | 2098 |
| La | 139 | 21.50 | 35.30 | 37.40 | 21.90 | 41.00 | 40.80 | 47.30 | 42.90 | 50.10 | 100 | 61 |
| Ce | 140 | 45.90 | 97.30 | 98.50 | 39.10 | 96.20 | 131.00 | 63.60 | 93.40 | 85.10 | 384 | 196 |
| Pr | 141 | 3.94 | 7.45 | 7.75 | 4.89 | 9.40 | 9.70 | 11.70 | 9.95 | 12.70 | 13 | 10 |
| Nd | 146 | 11.60 | 26.00 | 26.90 | 18.50 | 35.20 | 35.20 | 43.30 | 35.70 | 47.30 | 52 | 39 |
| Sm | 147 | 1.93 | 4.64 | 4.62 | 3.45 | 6.74 | 7.38 | 9.24 | 7.36 | 10.20 | 12 | 6 |
| Eu | 151 | 0.25 | 0.70 | 0.69 | 0.58 | 1.26 | 1.41 | 1.72 | 1.41 | 1.95 | 3 | 1 |
| Gd | 157 | 1.79 | 4.11 | 3.92 | 3.11 | 6.10 | 7.06 | 8.63 | 6.73 | 9.39 | 9 | 7 |
| Tb | 159 | 0.29 | 0.54 | 0.47 | 0.37 | 0.88 | 1.22 | 1.19 | 1.31 | 1.36 | 1 | 1 |
| Dy | 161 | 1.90 | 3.30 | 2.83 | 2.14 | 5.10 | 5.88 | 6.93 | 5.90 | 8.01 | 9 | 6 |
| Ho | 165 | 0.40 | 0.71 | 0.50 | 0.37 | 0.88 | 1.01 | 1.07 | 1.02 | 1.24 | 2 | 1 |
| Er | 166 | 1.37 | 1.87 | 1.60 | 1.07 | 2.54 | 2.55 | 2.80 | 2.55 | 3.26 | 18 | 20 |
| Tm | 169 | 0.25 | 0.32 | 0.27 | 0.15 | 0.38 | 0.36 | 0.39 | 0.36 | 0.46 | 1 | 0 |
| Yb | 172 | 1.79 | 2.25 | 1.89 | 1.00 | 2.44 | 2.28 | 2.40 | 2.22 | 2.81 | 4 | 3 |
| Lu | 175 | 0.32 | 0.38 | 0.33 | 0.17 | 0.40 | 0.34 | 0.35 | 0.33 | 0.40 | 1 | 0 |
| Hf | 180 | 2.81 | 3.46 | 3.63 | 0.89 | 2.77 | 0.80 | 0.84 | 0.98 | 1.14 | 4 | 8 |
| Ta | 181 | 1.66 | 1.72 | 1.82 | 0.45 | 1.21 | 0.32 | 0.35 | 0.01 | 0.02 | 0 | 0 |
| Pb | 208 | 24.80 | 26.90 | 24.30 | 11.40 | 36.10 | 27.80 | 23.20 | 1.65 | 0.64 | 200 | 709 |
| Th | 232 | 31.60 | 26.60 | 27.30 | 7.73 | 16.40 | 10.10 | 9.25 | 12.30 | 11.50 | 4 | 6 |
| U | 238 | 6.15 | 5.20 | 5.25 | 1.28 | 2.81 | 1.30 | 1.43 | 1.37 | 1.88 | 3702 | 129 |

Note: “/” means below limit of the determination.
